# Proteome-wide analysis of human motif-domain interactions mapped on influenza a virus

**DOI:** 10.1186/s12859-018-2237-8

**Published:** 2018-06-25

**Authors:** Carlos A. García-Pérez, Xianwu Guo, Juan García Navarro, Diego Alonso Gómez Aguilar, Edgar E. Lara-Ramírez

**Affiliations:** 1 Centro de Biotecnología Genómica, Instituto Politécnico Nacional, Reynosa, Tamaulipas Mexico; 2 Unidad de Investigación Biomédica de Zacatecas, Instituto Mexicano del Seguro Social, Interior Alameda # 45, Colonia Centro, CP. 98000 Zacatecas, Zac Mexico; 3 Oracle MDC, Guadalajara, Mexico

**Keywords:** Motif-domain interactions, Protein-protein interactions, Influenza, Data-mining

## Abstract

**Background:**

The influenza A virus (IAV) is a constant threat for humans worldwide. The understanding of motif-domain protein participation is essential to combat the pathogen.

**Results:**

In this study, a data mining approach was employed to extract influenza-human Protein-Protein interactions (PPI) from VirusMentha,Virus MINT, IntAct, and Pfam databases, to mine motif-domain interactions (MDIs) stored as Regular Expressions (RegExp) in 3DID database. A total of 107 RegExp related to human MDIs were searched on 51,242 protein fragments from H1N1, H1N2, H2N2, H3N2 and H5N1 strains obtained from Virus Variation database. A total 46 MDIs were frequently mapped on the IAV proteins and shared between the different strains. IAV kept host-like MDIs that were associated with the virus survival, which could be related to essential biological process such as microtubule-based processes, regulation of cell cycle check point, regulation of replication and transcription of DNA, etc. in human cells. The amino acid motifs were searched for matches in the immune epitope database and it was found that some motifs are part of experimentally determined epitopes on IAV, implying that such interactions exist.

**Conclusion:**

The directed data-mining method employed could be used to identify functional motifs in other viruses for envisioning new therapies.

**Electronic supplementary material:**

The online version of this article (10.1186/s12859-018-2237-8) contains supplementary material, which is available to authorized users.

## Background

Influenza viruses are negative-sense single-stranded RNA viruses that belong to the Orthomyxoviridae family. The influenza viruses are grouped in three types: A, B, and C. Among them, the Influenza A virus (IAV) is considered an infectious threat worldwide for humans [1]. The IAV genome is segmented, which allows the genetic exchange through reassortments within different hosts such as humans, poultry and swine. The IAV is classified into subtypes according to the surface proteins HA and NA in distinct strains. The strains most studied for affecting humans are the known pandemic H1N1, the avian origin strain H5N1, and the seasonal strains H3N2, H2N1, and H2N2. These IAV variants have been circulating in human population.

The viral cycle of IAV is developed within host cells where the virus produces gene information that is translated into ten functional proteins which interact with host proteins during the infectious process [2]. Studies on protein-protein interactions of IAV gene products have provided some interesting information in the context of virus-host interaction. For example, the study by Shapira et al., [3] provides three thought-provoking observations: 1) the influenza virus-virus protein interactions are highly interconnected, suggesting as feature related to the formation of a compact virion, 2) the influenza virus proteins interact with a higher number of human proteins than the human to human interactions, as a viral advantage to increase the heterogeneity of activity per protein, and 3) the human proteins contacted a greater number of influenza proteins, suggesting the possible presence of multiprotein complexes of virus-host interactions. Another study, using a statistical system biology approach, revealed that the inflammatory protein-protein interaction network for IAV is distinct to protein-protein interactions for normal processes (in human body). This information could be crucial for the understanding of the mechanism of pathogenesis to prevent and control the complications related to the infection [4].

The protein-protein interactions (PPI) can be classified into four classes: domain-domain interactions (DDIs), mutual fit interactions, induced fit interactions and linear motif-domain interactions (MDIs) [5]. Among these categories, the linear motif-domain interactions are preferentially employed for viruses. It could suggest that the viruses lack sequence similarity with human proteins, thus, viruses tend to compete with human proteins for domain binding sites that contain short linear motifs, i.e. motif-domain interactions [5]. In an evolutionary context, the viruses overcome the disadvantage of their small genome size by linear motif convergent evolution, which implies the motifs can appear in none related proteins or in different species [5, 6]. With these evolutionary mechanisms the viruses hijack or mimic host cellular processes successfully during the viral reproduction [5]. Therefore, the identification of the viral motifs that play a role in virus-host PPI context can help us to understand some of the viral cycle processes that are carried on the host cell. This kind of information can be useful to design new ways to prevent the viral infection or develop new antiviral treatments.

In the present study, human motif-domain interactions, reported for protein crystal structures, were searched on viral protein sequences from IAV strains. The obtained linear motif-sequences were analyzed for their conservation on a proteome-wide comparative analysis of five IAV strains. The MDIs functionality was further analyzed thorough gen ontology (GO) analysis. The results provide valuable information on host-viral interactions in the biology of the virus and could be used for devising rational therapy that targets IAV-human functional motifs.

## Methods

### IAV sequence collection

The IAV sequences were retrieved from the Virus Variation Resource of the National Center for Biotechnology Information [7]. The parameter options to retrieve the protein sequences were employed as follows. Virus Species = Influenza A. Subtype = H1N1, H5N1, H3N2, H1N2 and H3N2. Host = Human. Years = any. Complete sequences = only. Collapse similar sequences, this parameter allows to remove the similar sequences. The protein sequences were downloaded in fasta format and organized according to the protein and strain subtype as H1N1, H3N2, H1N2, H2N2, and H5N1, and to the proteins HA, NA, and so on. The number of sequences per protein and strain tested is shown in Table 1. A total of 51,242 IAV sequences isolated from human host were retrieved. More sequence information for H3N2 (*n* = 26,421) and H1N1 (*n* = 22,803) strains are available than the other strains, and more information for HA (*n* = 13,488) and NA (*n* = 10,015) than for other proteins. Sequences for H1N2 and H2N2 were < 100 for each protein (Table 1).

**Table 1:** The total number of sequence information retrieved for IAV

Protein	H1N1	H5N1	H3N2	H1N2	H2N2	Total
HA	6329	215	6831	33	80	13,488
NA	4196	184	5514	29	92	10,015
M1	621	37	426	6	26	1116
M2	593	56	701	9	23	1382
NS1	1735	130	2160	13	61	4099
NS2	550	71	459	8	26	1114
NP	1172	97	1286	8	69	2632
PA	2561	140	2664	12	67	5444
PB1	2260	123	2367	11	63	4824
PB1-F2	230	58	1360	6	36	1690
PB2	2556	155	2653	8	66	5438
Total	22,803	1266	26,421	143	609	51,242

### Data-mining of human motif-domain interaction data

**Fig. 1:**
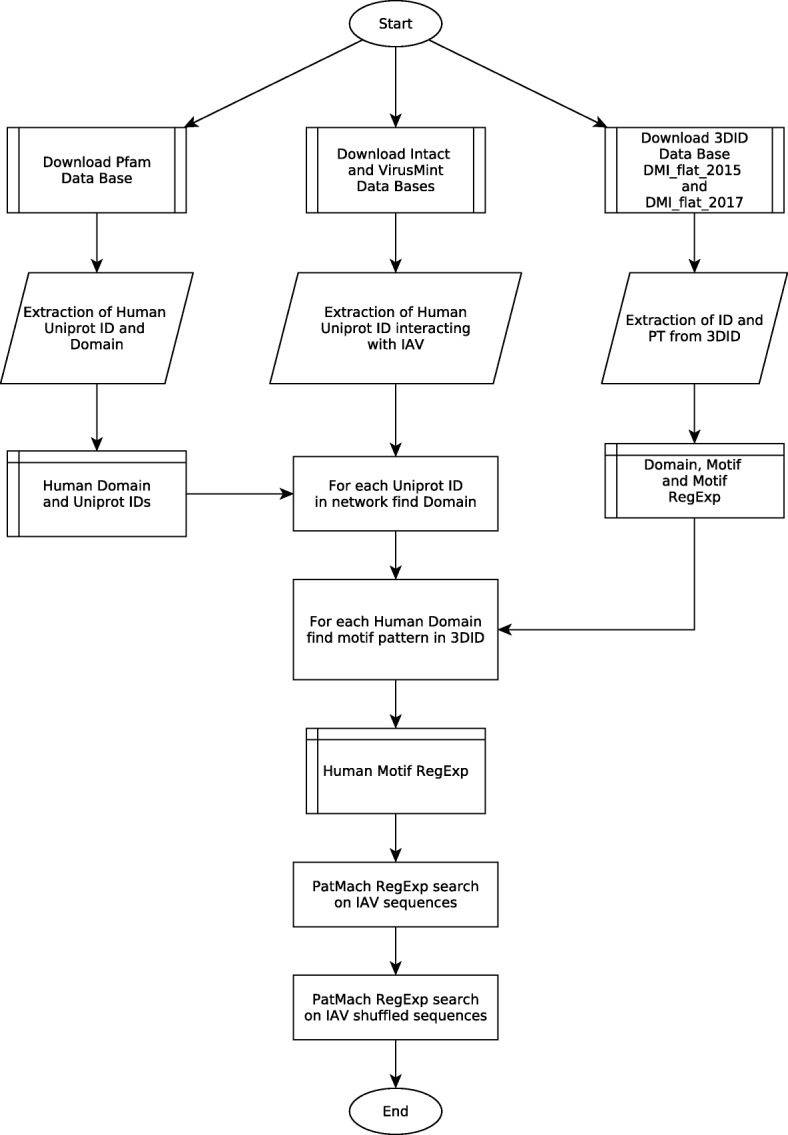
Flow diagram of the data mining methodology employed

The workflow for the MDIs extraction is depicted in Fig. 1. The Pfam database [8] was downloaded and mined to extract the human domain information based on the sequence UNIPROT identification code (UNIPROT ID). The IntAct [9] Virus MINT [10] and VirusMentha [11] databases were mined to extract information about influenza virus-human PPI network using the sequences matched with the UNIPROT ID. This information was queried for matching with the Pfam human protein domain database. The result was a series of UNIPROT IDs of human sequences related to human domain information associated with an influenza virus in a PPI network (Additional file 1: Table S1). This information was used for a final query on 3DID database [12], from which human MDIs were retrieved (Additional file 1: Table S2). 3DID is a website that contains information regards domain-domain and motif-domain interactions for which high-resolution three-dimensional structures are known. The human MDIs information was gathered as a Regular Expression (RegExp). RegExp was shown in a specific form, such as: G.{0,2}G.{0,2}K, indicating that the motif starts with the amino acid G, following 0 or 2 (represented in the brackets) any amino acids (represented by the dot “.”), then repeating with the G similar to the previous and terminating with K. If a square contains two or more amino acid letters (e.g [G,K]), it means that this position can be any of them (G or K). Those RegExp were used to feed the stand-alone version of the program PatMatch [13] in order to search linear sequence motifs on IAV proteins. The result was a linear amino acid sequence that matches with a RegExp for a human MDIs peptide as previously we did in [14]. The obtained information was further stored into an in house MySQL database (Additional file 2), which was consulted to perform the analyses about the motif information. The mysql database can be installed in any MySQL sever using the following command line “mysql -u root -*p* < new_influenza_motifs.sql”. In the MySQL database the sequence ID from virus variation was changed to UNIPROT IDs. The database can also be can be accessed via the following web link (http://visualanalytics.land/cgarcia/MotifSearch/index.html).

### Identification of potential functional of motifs

First, it was carried out a descriptive analysis based on the most frequent counts of human MDIs RegExp motifs that matched an amino acid sequence in protein of at least one strain. As some motifs are very short (3 amino acids) they could occur in a protein sequence by chance leading to a high false-positive rate. Hence in order to reduce the false-positive rate, we shuffled each of all our IAV protein datasets (Table 1) with the help of the protein shuffle online tool (http://www.bioinformatics.org/sms2/shuffle_protein.html) [15]. The shuffled protein datasets were then used to compare the motif matches in the original IAV sequences. We assumed that if frequently matched amino acids motif (> 70%) in the original IAV sequences is occuring rarely in the shuffled sequence dataset, it is most likely to be a functional motif [16]. The RegExp that matched more than 70% of the protein’s sequences in strain was further filtered [17]. The percentage of RegExp means the proportion of matched amino acid motif for a specific protein dataset. For example a total of 6329 HA sequences from H1N1 human strains were retrieved, thus, a RegExp with an occurrence of more than 70% for the HA protein, means that more than 4430 proteins of the total 6329 have a RegExp matched at an amino acid specific position. Finally, the search engine of the immune epitope database (IEDB) [18] was used to assess whether the amino acid motifs are part of experimentally reported epitopes. The parameters of query on IEDB were as follow: Epitope = Linear epitope. Option = Substring. Organism = Influenza A virus (ID:11320, influenza A).

### Gen ontology annotation and enrichment analysis of IAV-human network

The domains associated to the mapped human MDIs were annotated for their Gen Ontology (GO) related terms with the help of the 3DID search engine and the Pfam database. The obtained GO annotation for domains were summarize with the REVIGO online tool [19] to determine the biological processes. In addition the IAV-human network containing the mined information from the above mentioned databases was analyzed with the BiNGO tool [20] for Cytoscape [21]. BiNGO allows us to analyze the GO categories statistically overrepresented in the IAV-human network. The MCODE complement of Cytoscape was used to find highly interconnected regions (clusters) in the IAV-Human ontology network produced by BiNGO.

## Results

### Global comparative analysis of the mapped motifs on IAV viral proteins and strains

From 1093 interactions of the mined IAV-human network (Additional file 1: Table S1) the human sequences with domain information were used to mine the 3DID database. With this information a total of 110 RegExp (Additional file 1: Table S2) associated to human MDIs were retrieved from 3DID. Those RegExp were searched on the IAV viral proteins from five strains to map an amino acid motif related to a human MDIs. The total number of RegExp that map the MDIs in the entire proteome from five strains was of 1007 matches (Table 2). The number of RegExp matches was higher for the H1N1 and H3N2 strains. Seven proteins (HA, NA, NS1, NP, PA, PB1; PB2) showed the higher number of RegExp matches (Table 2). The number of MDIs that matched an amino acid motif sequence in more than 70% of the protein was lower that the motifs that matched less than 70%. Interestingly the number of motifs > 70% was higher in ribonucleoproteins (NP, PA, PB1, PB2) than in the immunodominant proteins (NA, HA). This observation suggests that functional motifs are more stable in the proteins involved in the transcriptional machinery of the IAV. On the other hand, the fact that the number of motifs with an occurrence of less than 70% is higher than the motifs with an occurrence above of 70%, suggests that those rarely motifs could be arising by mutations and are present in specific IAV strains as previously indicated [22]. Most of those motifs mapped an amino acid sequence that were absent or occurred very rarely (particularly those MDIs of 3 to 4 amino acid of length) in the shuffled datasets indicating that they are functional motifs.

**Table 2:** Comparison of the Motif counts in the five IAV strains per proteins

strain	H1N1		H5N1		H3N2		H1N2		H2N2		
Occurrence	> 70%	< 70%	> 70%	< 70%	> 70%	< 70%	> 70%	< 70%	> 70%	< 70%	Total
HA	5	46	8	15	5	37	10	5	3	9	143
NA	3	41	3	15	8	28	8	7	5	4	122
M1	2	13	2	1	2	6	2	0	2	0	30
M2	1	11	2	0	1	12	1	0	2	0	30
NS1	9	29	8	9	6	25	7	2	7	2	104
NS2	1	11	2	5	1	8	1	0	1	1	31
NP	7	23	9	3	7	24	7	5	8	1	94
PA	12	33	15	13	14	25	14	1	15	4	146
PA-X	3	14	4	3	3	19	2	1	2	3	54
PB1	14	22	15	10	14	20	14	2	15	1	127
PB1-F2	0	7	2	4	2	12	1	3	1	2	34
PB2	9	18	9	3	9	20	9	1	9	5	92
Total	66	268	79	81	72	236	76	27	70	32	1007

### Comparative analysis of the most frequently motifs on the viral proteins and strains

**Fig. 2:**
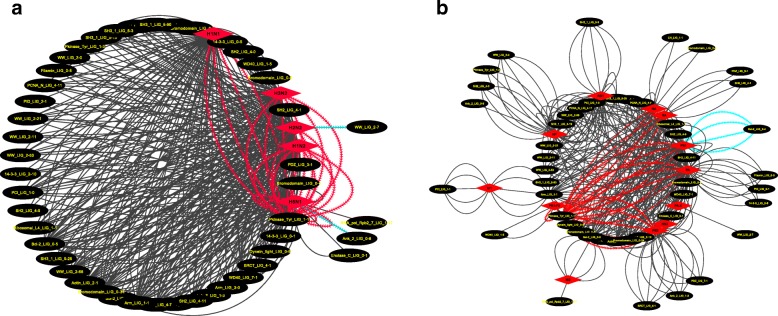
Circular layout network representation for MDIs mapped across the five strains and proteins. **a**) Strain-MDIs network **b**) Protein-MDI network. The red diamond represents strains **a**) and proteins **b**) respectively, the black ellipse represents the MDIs. The edges highlighted are the motifs mentioned in the text. Both figures can be reproduced in Cytoscape with the first four column information presented in the Additional file 2: Table S3

Humans can be infected by several IAV strains. To understand how motif compositions have been diverged among the several known human pathogenic IAV strains we performed a more in-depth comparative analysis among the five strains and proteins in the potential functional MDIs with an occurrence above of 70% (Additional file 1: Table S3, Fig. 2).

Most of the motifs were mapped across the five strains (H1N1, H5N1, H3N2, H1N2, H2N2) strains to some extent. For example, the widely conserved Pkinase_Tyr_LIG_1–1 motif, was found in the five strains (Fig. 2a, highlighted red edges). However, a few exclusive motifs were also observed for particular strains. For example, the motif WW_LIG_2–7 for the H2N2 strain or the Ank_2_LIG_0–8 for the H5N1 strain (Fig. 2a, highlighted blue edges). Those motifs were also mapped across several virus proteins comprising the entire proteome, for example the same motif Pkinase_Tyr_LIG_1–1 motif was shared by six proteins HA, NA, PA, PB1, PB1-F2, PB2 (Fig. 2b, highlighted red edges). But exclusive MDIs for specific proteins also exist, for example the motif Bcl-2_LIG_2–4 for PB1 (Fig. 2b, highlighted blue edges).

**Fig. 3:**
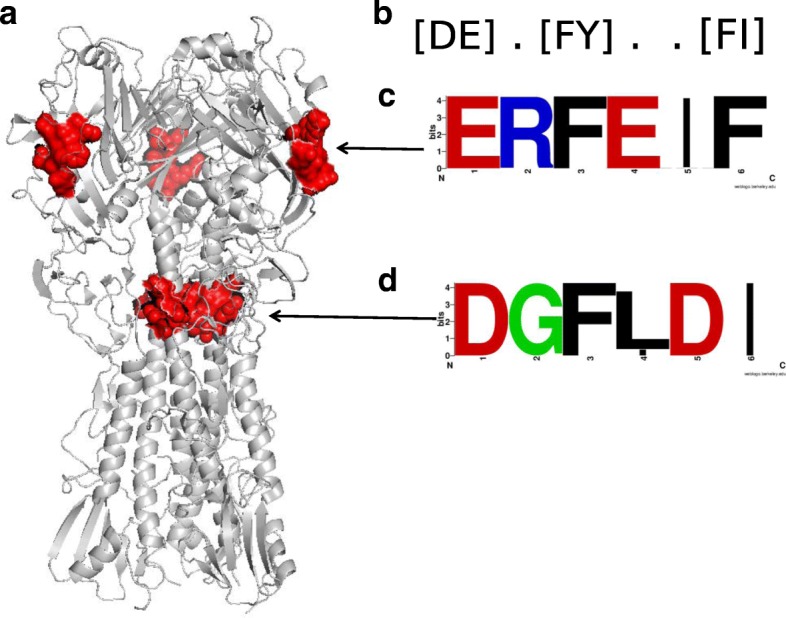
The structural localization for the Pkinase_Tyr_LIG_1–1 MDIs on the HA and the amino acid motifs for the H1N1 strain. **a**) The Haemagglutinin (HA, PDBID: 3Al4), with the Pkinase_Tyr_LIG_1–1 MDI marked in red. **b**) The RegExp, **c**, **d**) Seqlogo showing the frequently amino acid motifs found in the H1NI strain

The amino acid short sequences mapped with the RegExp revealed that the motifs had a few amino acid variants (Additional file 1: Table S3). The motif amino acid sequences are quite similar for the NS1, NS2, NP, PA, PB1, PB2, but not on the HA and NA proteins. Moreover, some MDIs can be located more than one time within the protein sequences (Additional file 1: Tables S3 and S4). For example, in the protein HA of H1N1 there are two positions (129–134 ERFEIF and 430–435 DGFLDI) for the Pkinase_Tyr_LIG_1–1 (RegExp [DE].[FY]..[FI]) MDIs (Fig. 3, Additional file 1: Table S4). As motifs are also potential epitopes [6] we evaluate whether the amino acid motifs that we obtained were immune epitopes. To do so, we used IEDB and indeed some our motifs were parts of reported epitopes confirmed by experiments (Additional file 1: Table S4). MDIs related to phosphorylation processes, chromatin remodelling, cytoskeleton rearrangements were associated with those epitopes.

### Gen ontology analysis of the MDIs and human-IAV network

**Fig. 4:**
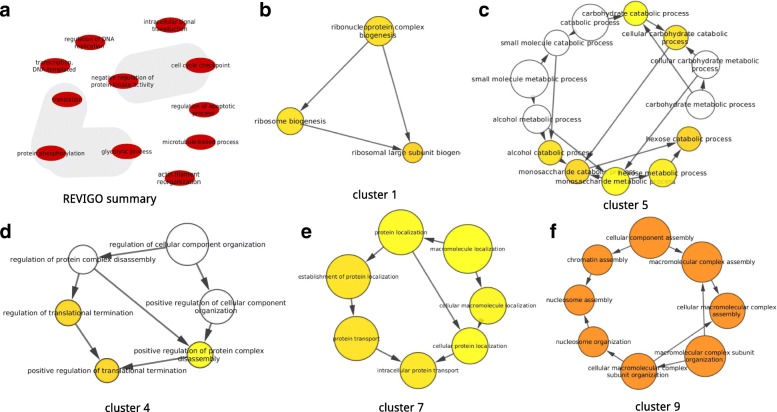
Biological processes related to the MDIs and GO clusters of the IAV-Human mined Network. **a**) REVIGO summary for the MDIs related GO terms **b**, **c**, **d**, **e**, **f**) Identified GO clusters in the IAV-human Network related to the MDIs with the MCODE app for cytoscape

The above described MDIs have distinct functionalities (Additional file 1: Table S5). For example, the Actin_LIG_2–1 motif-domain is involved in interaction with the cytoskeletal architecture of the cell. The Ank_2_LIG_1–3 motif-domain is involved in the mediation of varied PPI and is very common in known proteins. These functionalities were also evident in the ontology analyses where the motifs were found related to essential biological processes such as microtubule-based processes, regulation of cell cycle check point, regulation of replication and transcription of DNA, etc. (Fig. 4a). More interestingly in the ontology analyses for our mined IAV-human network were connected with some MDIs biological processes (Fig. 4b-f). For example, for the transcription DNA-templated and translation MDIs biological process were also identified GO clusters related such as ribosome biogenesis and regulation of translational processes in the IAV-Human network analysis (Fig. 4 b,d), etc. This observation suggests that our rational directed data-mining method could be useful to identify human MDIs in other virus pathogens.

## Discussion

In this work, we used RegExp associated to human motif-domain interaction (host-like) to identify potential functional motifs present on the IAV proteins (Additional file 1: Table S4). The identification of linear motif is a difficult task due to their small size, the large number of motifs in human proteins (more than one million motifs), and the evolutionary plasticity [23]. Some databases have been created to help in the identification of motifs. One of them is the Eukaryotic Linear Motif resource (ELM) [24], a database which is based on RegExp experimentally validated and curated from the literature. This database have been tested for the identification of conserved motifs on wide range viral proteins, and it was found that viruses use host-like peptide motifs as a widespread manner to mediate host-virus interactions that helps in the viral replication [16]. Another recent study uses the same database to apply filtering methods for predicting motif PPI between human and HIV-1 viruses. They found that motifs are located in disordered protein regions [25]. Another database is the 3DID which is also based on RegExp motifs, but it contains motif information classified in Domain-Domain Interactions (DDIs) and Motif-Domain Interactions (MDIs) identified from molecular interactions of protein structures deposited in the Protein Data Bank. The 3DID database was used in a recent study [26] to predict Grass carp annotated domains associated to a MDIs obtained from 3DID and ELM that were mapped on GCRV virus proteins. In contrast to those previous works, the present study employed a more rational directed data-mining methodology. The first step is to get an IAV-human network from mined information obtained from public databases related to virus-host interaction. The final step is to mine the 3DID database focusing in the obtaining of human MDIs. As a result we obtained MDIs associated to human hosts in RegExp forms. These MDIs were mapped in a IAV proteome from five human infecting strains and it was found 46 frequent human related MDIs that could participate in PPI with human proteins. Those functional motifs tend to appear less frequently immunodominant proteins (NA, HA) that in ribonucleoproteins (NP, PA, PB1, PB2) which suggest that motifs in ribonucleoproteins are essential motifs required for adaptation to the human host, because those proteins are related to replication processes of the virus. Moreover, the number motifs with an occurrence of less than 70% was higher in the immunodominant proteins which suggests that those rare motifs could be arising by mutations and are present in specific IAV strains. In general, the MDIs were mapped very frequently across the different viral proteins and the different IAV strains implying that this phenomenon is a result of convergent evolution by the IAV strains are mimicking to achieve the adaptation to the human host.

Those potential functional MDIs were related to essential biological processes for human cells such as microtubule-based processes, regulation of cell cycle check point, regulation of replication and transcription of DNA, etc., which are also required for the interactions of virus with their host [27–33]. For example, the Actin_LIG_2–1 and Dynein MDIs that are related to the family of cytoskeletal proteins, involved in the transportation of proteins within cells. Some reports indicated that viruses acquired those mimic mechanisms to manipulate the cytoskeleton to move within the host cell to be successful during the infection process [31]. In fact, a recent experiment to test the inhibition of Actin-myosin network formation on IAV infection and showed to be effective on the virus survival [34]. Moreover the motor protein Dynein participated in the translocation of the viruses to the perinuclear region and is also engaged in the intermittent movement of the viruses in interaction with the microtubules [30]. The above cited studies did not mention any specific amino acid motif participating on those virus-host interaction processes. The amino acid motif identification is crucial to identify the molecular mechanism by which the viruses interact with the protein of their hosts. For example, an experimental study in H5N1 strains identified the amino acid sequence ESEV on NS1 protein as a virulent factor that binds with PDZ proteins present in the respiratory epithelium of mammalian hosts [35]. Since epitopes on viral proteins are also functional motifs for the interactions of viruses with human host, IAV could use the similar mechanism for immune interaction with human, and IEDB can be utilized to check the occurrence of viral motif epitopes. For example the RegExp [DE].[FY]..[FI] (Pkinase_Tyr_LIG_1–1) on the HA of H1N1 allows to identify the amino acid sequence of the ERFEIF motif, which is part of 22 experimental reported T cell epitopes on the IEDB. In this context it was known that the adaptive human immune system detects conserved immunogenic sequences from a wide range of pathogens [6, 36]. But was also known that viruses scape from the adaptive immune system by decreasing immunogenic sequence motifs from their proteome [6, 37] However, we found evidence that IAV use linear epitope motif mimicry as a manner to circumvent the immune system. As our used data were obtained from experiments, the results derived are convincing and our directed data-mining method is proper to apply to other virus species.

## Conclusion

In conclusion, the present study we showed that 46 MDIs were harbored on IAV virus proteome of five strains. The MDIs resemble host-like mechanism, which is related to the virus survival within the host cell. The MDIs are part of immune epitopes, indicating the presence of such interactions. Thus, although the human-human and humans-virus interactions could be different, our analysis methodology uses databases validated by experiments making our more convincing and provides a strategy to apply in other unknown virus proteins, and could be used to design new therapies that targets those IAV-human functional motifs.

## Additional files

Additional file 1: **Table S1.** The IAV-human network mined from the Intact, Virus Mint and virus Mentha; **Table S2.** The 110 RegExp asociated to human motif-domains interactions mined from 3-DID; **Table S3.** The amino acids motifs matched with the RegExp associated to human MDIs; **Table S4.** The amino acids motifs that matched an epitope in the immune epitope database; **Table S5.** The GO annotation and function of the 46 most frequently Motifs. (XLSX 979 kb)

Additional file 2: The MySQL database. (TAR 71770 kb)
